# Brainstem Cavernous Malformation Mimicking Glioma in Neurofibromatosis Type 1: A Rare Diagnostic Pitfall

**DOI:** 10.3390/diagnostics16193090

**Published:** 2026-09-24

**Authors:** Arturs Laganovskis, Julija Dolgopolova, Cenk Eraslan, Karina Biserova, Ilze Strumfa, Marta Pūpola, Arturs Balodis

**Affiliations:** 1 Faculty of Medicine, Riga Stradins University, 16 Dzirciema Street, LV-1007 Riga, Latvia; 2 Department of Neurosurgery, Pauls Stradins Clinical University Hospital, 13 Psonu Street, LV-1002 Riga, Latvia; 3 Department of Radiology, Ege University, 35040 Bornova, Turkey; 4 Institute of Pathology, Pauls Stradins Clinical University Hospital, 13 Pilsonu Street, LV-1002 Riga, Latvia; 5 Department of Pathology, Riga Stradins University, 16 Dzirciema Street, LV-1007 Riga, Latvia; 6 Department of Radiology, Riga Stradins University, 16 Dzirciema Street, LV-1007 Riga, Latvia; 7 Institute of Diagnostic Radiology, Pauls Stradins Clinical University Hospital, 13 Pilsonu Street, LV-1002 Riga, Latvia; 8 Faculty of Medicine and Life Sciences, University of Latvia, LV-1004 Riga, Latvia

**Keywords:** cavernous malformation, glioma, neurofibromatosis type I, susceptibility-weighted imaging

## Abstract

**Background and Objectives:** A cerebral cavernous malformation (cavernoma) is a lesion composed of clustered abnormal capillary channels lacking normal brain parenchyma between them. Most cerebral cavernomas are sporadic but up to 20% have familial autosomal dominant inheritance pattern. They are extremely rare, with an annual incidence of approximately 0.15–0.56 per 100,000 population. The treatment ranges from observation for asymptomatic patients to surgical resection. **Case Description:** A 20-year-old patient with type I neurofibromatosis (*NF1*) and bilateral optic pathway gliomas since infancy underwent radiotherapy at age seven for an enlarging right medullary lesion, radiologically diagnosed as a glioma. In 2025, he developed intermittent headaches, limited cognitive function, and altered speech. MRI in July 2025 showed enlargement, contrast enhancement, and a cystic component of the right medullary lesion with fourth ventricle compression. Suboccipital craniotomy, performed for presumed medullary glioma, revealed a hemorrhagic vascular lesion; histopathology confirmed a cerebral cavernous malformation and excluded glioma. Because the lesion predated radiotherapy and the availability of susceptibility-sensitive imaging in 2012 is uncertain, its relationship to irradiation cannot be established. The case therefore illustrates a pre-existing or occult cavernous malformation with subsequent radiological evolution. The postoperative course was uneventful, and the patient remains clinically stable. **Conclusions:** It is possible for cerebral cavernous malformations to mimic gliomas on imaging, particularly in patients with pre-existing CNS lesions such as type I neurofibromatosis. Susceptibility-weighted imaging (SWI) together with post-contrast T1-weighted imaging may aid in distinguishing vascular malformations from primary brainstem tumors.

## 1. Background

Neurofibromatosis I (*NF1*), also called Von Recklinghausen disease, is an inheritable neurocutaneous disorder and a phakomatosis that manifests by developmental changes in the nervous system, bones and skin. It has an autosomal dominant inheritance pattern. The *NF1* gene encodes neurofibromin. Neurofibromin is expressed in various tissues. It inhibits the RAS pathway. Mutations in the *NF1* gene lead to loss of neurofibromin expression. This promotes the development of neurofibromas, which contain Schwann cells, perineural cells, mast cells and fibroblasts. They affect the skin and arise from the peripheral nerves, along with their supporting network, which includes neurilemmal cells [1,2,3].

Optic gliomas occur in approximately 15–20% of patients suffering from *NF1*. These tumors are usually asymptomatic and remain so throughout life. Compared to sporadic cases, *NF1*-associated optic pathway gliomas generally exhibit a more favorable clinical course, frequently demonstrating prolonged stability or only indolent progression. The symptoms tend to appear prior to age six and present as visual acuity loss, strabismus, or proptosis, though adult or late childhood onset remains possible [2]. Brainstem gliomas, however, represent the second most prevalent central nervous system malignancy in patients with *NF1*, manifesting in roughly 4% of this patient population. In radiological evaluations, these neoplasms can be differentiated from classic T2-hyperintense *NF1*-associated hamartomas by their characteristic focal or diffuse enlargement of the brainstem, associated mass effect, and inconsistent contrast enhancement. Like optic gliomas, these tumors are seldom biopsied due to the high risk of inducing iatrogenic brainstem dysfunction [4].

Cerebral cavernous malformations are abnormally large clusters of “low flow” capillaries without any intervening brain parenchyma between the sinusoidal vessels. Usually they are surrounded by hemosiderin deposits and gliosis because of the periodical microhemorrhages and thrombosis. While most cavernomas are sporadic, approximately 20% of cases are inherited in an autosomal dominant fashion. The incidence of cerebral cavernous malformations is about 0.4% to 0.8%. They differ in size from under a millimeter to several centimeters and may occur anywhere in the central nervous system, with up to 20% located in the brainstem. The symptoms of this specific lesion manifest as intermittent headaches, seizures, focal neurologic deficits, or symptomatic hemorrhage [5]. It is important to add that there is a growing body of literature supporting a significant correlation between prior cranial radiation therapy and the sequential development of cavernous malformation within the irradiated tissues [6,7]. Susceptibility-sensitive sequences play a pivotal role in radiological differentiation via MRI. They are recommended for the investigation and follow-up of cerebral cavernomas. On imaging, these lesions typically exhibit a characteristic “popcorn” or “berry” appearance, delineated by a peripheral rim of signal loss secondary to hemosiderin deposition [8].

In this article, we present a rare case report of a patient with neurofibromatosis type 1 whose cavernous malformation was initially misdiagnosed as a brainstem glial tumor. This clinical case highlights the importance of comprehensive radiological differential diagnosis, particularly in patients with neurofibromatosis type I and other inherited phakomatoses, where the presence of characteristic disease-associated lesions may create a diagnostic bias toward expected tumor manifestations, potentially overlooking other unrelated pathologies. It also demonstrates the atypical evolution of a lesion located in the brainstem. This case highlights that, in patients with *NF1*, an enlarging brainstem lesion with susceptibility-related blooming may represent a cavernous malformation rather than glioma, particularly when prior cranial irradiation complicates radiologic interpretation.

## 2. Case Report

A 20-year-old patient with neurofibromatosis type I was diagnosed at the age of one year and two months after evaluation for exophthalmos in the right eye, when brain MRI revealed bilateral optic pathway gliomas involving the optic nerves and chiasm. Since then, the patient had undergone regular follow-up by ophthalmologists and neurologists.

According to the mother, clinical deterioration was noted in 2010 when frequent seizures, pain in the orbital region and progression of the listed lesions led to multidisciplinary oncologic evaluation and initiation of chemotherapy. From October 2010 to February 2012, the patient received six cycles of actinomycin/vincristine-based therapy. This stabilized tumor growth, however, optic nerve atrophy of the right eye had already developed, while vision in the left eye was preserved.

On MRI performed on March 2012, enlargement of a lesion in the medulla oblongata was observed and the lesion was interpreted radiologically as a glioma (Figure 1). The available clinical and imaging documentation does not permit a precise assessment of whether the patient had objective neurological deterioration attributable to this lesion at that time. Nevertheless, the combination of interval enlargement, the patient’s known *NF1*-associated CNS pathology, and the perceived risk of continued brainstem progression led the multidisciplinary team to treat the lesion as a presumed brainstem glioma. Susceptibility-sensitive imaging, including GRE/T2*-weighted imaging and SWI, was not available in the archived MRI examination from 2012. Based on the historical imaging protocol used at that time, such sequences were not routinely available or included in the examination. The absence of a reported hemorrhage on conventional MRI does not exclude an occult cavernous malformation, because susceptibility-sensitive imaging may be required to demonstrate hemosiderin and blood products. Consequently, radiotherapy was initiated for a presumed brainstem glioma. From April to June 2012, the patient received cranial radiotherapy to the posterior fossa, targeting the lesion in the medulla oblongata, to a total dose of 54.8 Gy delivered in 30 fractions of 1.6–1.8 Gy. The treatment was intended to control the interval growth of the presumed glioma.

The subsequent course was further complicated by hydrocephalus which required ventriculoperitoneal shunt insertion in 2015. Two years later, the patient developed recurrent neurological deterioration with aphasia and gaze palsy due to shunt dysfunction and recurrent hydrocephalus, for which repeat surgery was performed. Afterward, shunt function remained stable without major further decline.

At an outpatient reassessment in 2025, the patient reported intermittent headaches that had not significantly worsened over time, as well as increasing numbers of cutaneous neurofibromas in recent years. Cognitive function was limited and speech was altered, hypothetically attributable either to prior radiotherapy or to progression of the medullary lesion; the performance status was ECOG 1. On examination, the general condition was relatively satisfactory, with multiple cutaneous and scalp neurofibromas, longstanding abnormal positioning of the right eye, horizontal nystagmus bilaterally, and an unstable sensitized Romberg test without dysmetria.

MRI performed on September 2023 showed *NF1*-associated multifocal intracranial pathology, including lesions in the right medulla oblongata, corresponding to the lesion whose enlargement was first identified in 2012; tectal/inferior collicular region with occlusion of the cerebral aqueduct, bilateral optic pathway gliomas with prechiasmatic optic nerve involvement, and a lesion in the right globus pallidus, in addition to cervical plexiform neurofibromas and multiple subcutaneous neurofibromas (Figure 2). Follow-up MRI on February 2025 showed stable gliomas in the right globus pallidus, tectum mesencephali, and optic pathways. However, there was a slight interval enlargement of the lesion in the right medulla oblongata compared with September 2023.

Because of this change, an earlier follow-up MRI was recommended and performed in July 2025. This examination demonstrated further enlargement of the dorsal right medullary lesion, increasing from approximately 13 mm in 2023 to 17 mm in 2025, with heterogeneous contrast enhancement. These findings initially reinforced suspicion of brainstem glioma (Figure 3). The lesion was measured 17 × 15 × 27 mm and had a complex structure with a marginal cystic component directed toward the fourth ventricle, causing compression of the ventricular lumen. Susceptibility-weighted imaging showed marked blooming corresponding to hemosiderin deposition within the lesion, but this was interpreted as a post-radiotherapy effect rather than a vascular malformation (Figure 4).

Following the radiological progression and the presumed diagnosis of medullary glioma, an elective suboccipital craniotomy with subtotal lesion resection was performed in October 2025. Intraoperatively, however, a hemorrhagic vascular lesion was identified instead of an intrinsic glioma. The lesion was carefully dissected, and its vascular channels were coagulated. There were no additional abnormal vessels. The patient’s clinical condition improved after surgery: headaches resolved and speech improved. The MRI performed in February confirms the success of the operation, demonstrating that a significant part of the cavernoma, including the cyst, was extirpated (Figure 5). Follow-up MRI is planned in September 2026, with subsequent examinations adjusted according to stability. Each examination should include high-resolution T2-weighted and 3D FLAIR sequences, pre- and post-contrast 3D T1-weighted MPR imaging when clinically indicated, DWI/ADC nat, PWI and a susceptibility-sensitive sequence, preferably SWI. The residual lesion will be assessed for interval growth, new hemorrhage, increasing cystic change, mass effect, or new enhancement. Histopathological examination revealed groups of flattened vascular channels (Figure 6) that were lined by immunohistochemically detectable, morphologically plain endothelium (Figure 7) but lacked properly organized smooth muscle and elastic layers, as expected in a cavernous malformation. Focal microcalcifications were present. The lesion was devoid of intervening brain tissue. There was no evidence of glial hypercellularity, mitotic activity, Rosenthal fibers, nuclear palisades, hyperchromasia, necrosis, microvascular proliferation or inflammation. The proliferative activity by Ki-67 protein expression was low (Figure 8). Thus, the morphological findings confirmed the diagnosis of a cerebral cavernous malformation. Considering the non-neoplastic vascular origin of the focal pathological lesion, somatic genetic testing for gliomas was not indicated (Figure 9).

## 3. Discussion

This case illustrates a rare diagnostic pitfall in *NF1*. Given the patient’s long history of CNS lesions, the enlarging medullary lesion was interpreted as a brainstem glial tumor. Intraoperatively, however, it proved to be a cavernous malformation. Patients suffering from *NF1* are predisposed to intracranial gliomas, particularly optic pathway and brainstem gliomas, which makes new or enlarging brainstem lesions especially prone to being interpreted as gliomatous in origin. In this case, the coexistence of multiple *NF1*-related lesions strengthened this assumption [9].

There is a wide range of differential diagnoses that should be considered during the histological evaluation of an intracranial mass lesion in *NF1* patients. Most frequently, these patients develop low-grade gliomas, which are located in the optic pathway or hypothalamus (66–75%), followed by the brainstem (10–15%) [10]. The predominant location in the optic tract has been associated with light-induced neuronal activity. In a mouse model, retinal neurons harboring *NF1* mutation released increased amounts of neuroligin 3 into the optic nerve, thus promoting tumorigenesis; blockade of this molecule or light deprivation at a certain age abrogates tumor formation [11].

Due to the mostly indolent course, as well as the location of the *NF1*-related tumors, the usual clinical approach includes either follow-up or chemotherapy. Surgical treatment or biopsy is not always performed, limiting the morphological data on the tumor spectrum in *NF1*. However, it is generally presumed that most of low-grade gliomas in *NF1* are pilocytic astrocytomas which represent at least 85% of all *NF1*-related pediatric brain tumors. Grossly, pilocytic astrocytomas are soft and gray—an appearance that is noticeably different from the hemorrhagic tissues, found intraoperatively in the given case. Cystic change is frequent in pilocytic astrocytoma, and the tumor can present as a cyst with a gray, soft mural nodule in the cyst wall [10].

On microscopic examination, pilocytic astrocytomas feature low or moderate cellularity and biphasic architecture of compact, dense areas, alternating with loose, myxoid, microcystic foci. The dense tissues consist of elongated pilocytic cells with bland nuclei and bipolar, “hair-like” processes which serve as the basis for the tumor’s name [12]. Brightly eosinophilic, corkscrew-shaped, elongated or beaded Rosenthal fibers are a classic component [12,13,14]. Pilocytic astrocytomas generally exhibit thin-walled blood vessels of the capillary type. However, hyalinization can lead to remarkable thickening of capillary walls and narrowing or even closure of the lumina. Glomeruloid microvascular proliferation is rare [12,15].

In our patient, a completely different tissue structure was observed. The whole lesion consisted of distended, thin-walled vascular channels that were lined by endothelium but lacked normal histological structure, without the proper architecture of smooth muscle and elastic membranes. The lesion was devoid of intervening brain tissue. Thus, the morphological picture was diagnostic of cerebral cavernous malformation [16,17] and virtually exclusive of glial tumor, including also pediatric diffuse low-grade gliomas [18,19], diffuse astrocytoma [20,21], glioblastoma [20,21] and pediatric diffuse midline high-grade glioma [18,20,21].

The lesion demonstrated interval growth, heterogeneous enhancement, and a cystic component, which supported the initial radiological suspicion of glioma (Figure 3 and Figure 4). However, hemorrhagic products and hemosiderin can make cavernomas look aggressive or tumor-like, and without susceptibility-sensitive sequences, they may be difficult to recognize preoperatively [7]. The differential diagnosis of an enlarging, hemorrhagic, and enhancing medullary lesion includes cavernous malformation, brainstem glioma, hemorrhagic metastasis, capillary or other vascular malformation, developmental venous anomaly with an associated cavernoma, subacute hematoma, and less commonly abscess or inflammatory lesions. In retrospect, several imaging features favored cavernous malformation over glioma, including marked susceptibility blooming on SWI, a peripheral hemosiderin rim, heterogeneous internal signal reflecting blood products of different ages, and a complex “popcorn” or “berry” appearance (Figure 4) [8]. SWI or GRE sequences are therefore key for identifying hemosiderin and blooming, and they may help distinguish vascular malformations from intrinsic brainstem tumors. This is particularly important in brainstem lesions, where imaging interpretation directly influences treatment planning [22].

The initial interpretation was also influenced by diagnostic anchoring in the setting of *NF1*. The patient had several distinct *NF1*-associated or *NF1*-related abnormalities, including optic pathway gliomas, a tectal/inferior collicular lesion, FASI, plexiform neurofibromas, and cutaneous neurofibromas. These entities should not be treated as interchangeable. Optic pathway and tectal lesions may represent gliomas; FASI is a separate, typically non-neoplastic imaging finding; and the medullary lesion ultimately proved to be a vascular malformation. This distinction is clinically important because attributing every new T2/FLAIR abnormality in a patient with *NF1* to glioma may delay recognition of a hemorrhagic vascular lesion [23].

Prior cranial radiotherapy is an important consideration in this setting. Radiation-induced cavernous malformations are a recognized late complication of childhood cranial irradiation. However, the chronological sequence in this patient did not permit the lesion to be classified straightforwardly as radiation-induced: the medullary lesion was already visible and had enlarged before radiotherapy was administered in 2012. An occult cavernous malformation could not be excluded even though GRE or SWI was not performed at that time. Conventional T1-, T2-, FLAIR-, and diffusion-weighted sequences may fail to demonstrate the full susceptibility pattern of a small or early lesion. The subsequent enlargement, hemorrhagic evolution, enhancement, and cystic change may represent the natural evolution of a pre-existing cavernous malformation, radiation-associated modification of such a lesion, or less likely a newly developed vascular malformation that arose within or adjacent to the pre-existing abnormality. The available imaging and pathological findings cannot distinguish these possibilities definitively. The available data do not demonstrate transformation of a glioma into a cavernous malformation. Instead, the lesion may have been a pre-existing or occult cavernous malformation that subsequently evolved radiologically; radiation-associated modification cannot be excluded, but a direct causal relationship remains unproven [6,7].

Symptomatic or enlarging brainstem cavernomas may require surgical treatment, especially when they are associated with hemorrhage or compression of adjacent neurovascular structures [4]. In this patient, the lesion compressed a ventricle and contained a cystic component, supporting the decision for surgical intervention. The intraoperative finding ultimately changed the diagnosis and underscores the importance of including vascular malformations in the differential diagnosis of brainstem masses. In patients with NF-1, especially those with prior cranial irradiation, a progressively enlarging brainstem lesion should prompt consideration of cavernous malformation, as this may alter both surgical planning and management.

## 4. Conclusions

Brainstem cavernous malformations can mimic intrinsic gliomas on neuroimaging and should remain in the differential diagnosis of enlarging brainstem lesions, particularly in patients with *NF1* and complex pre-existing CNS pathology. Susceptibility-sensitive MRI sequences, such as SWI or GRE, may help raise suspicion for a vascular malformation before surgery. In this case, the lesion was present before radiotherapy; therefore, the available evidence supports a cavernous malformation with subsequent radiological evolution, while a radiation-associated contribution remains possible but unproven. When a small residual lesion remains after surgery in the eloquent brainstem, management should include a documented MRI surveillance plan with susceptibility-sensitive imaging.

## Figures and Tables

**Figure 1:**
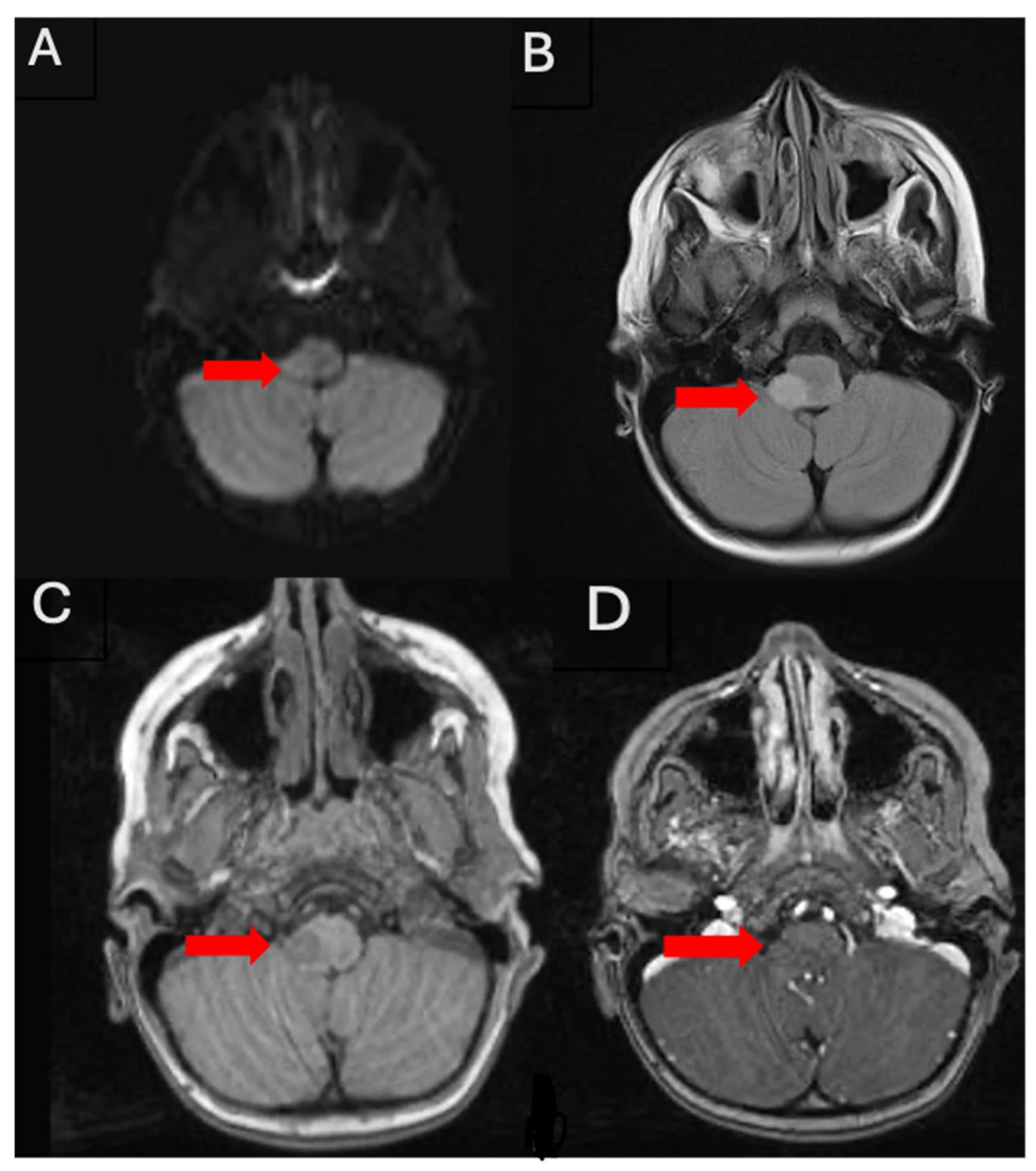
Lesion in the medulla oblongata (red arrow). Axial MRI images: (**A**) DWI, (**B**) FLAIR, (**C**) T1-weighted pre-contrast, and (**D**) T1-weighted post-contrast. There is no evidence of hemorrhage, no mass effect, and no contrast enhancement.

**Figure 2:**
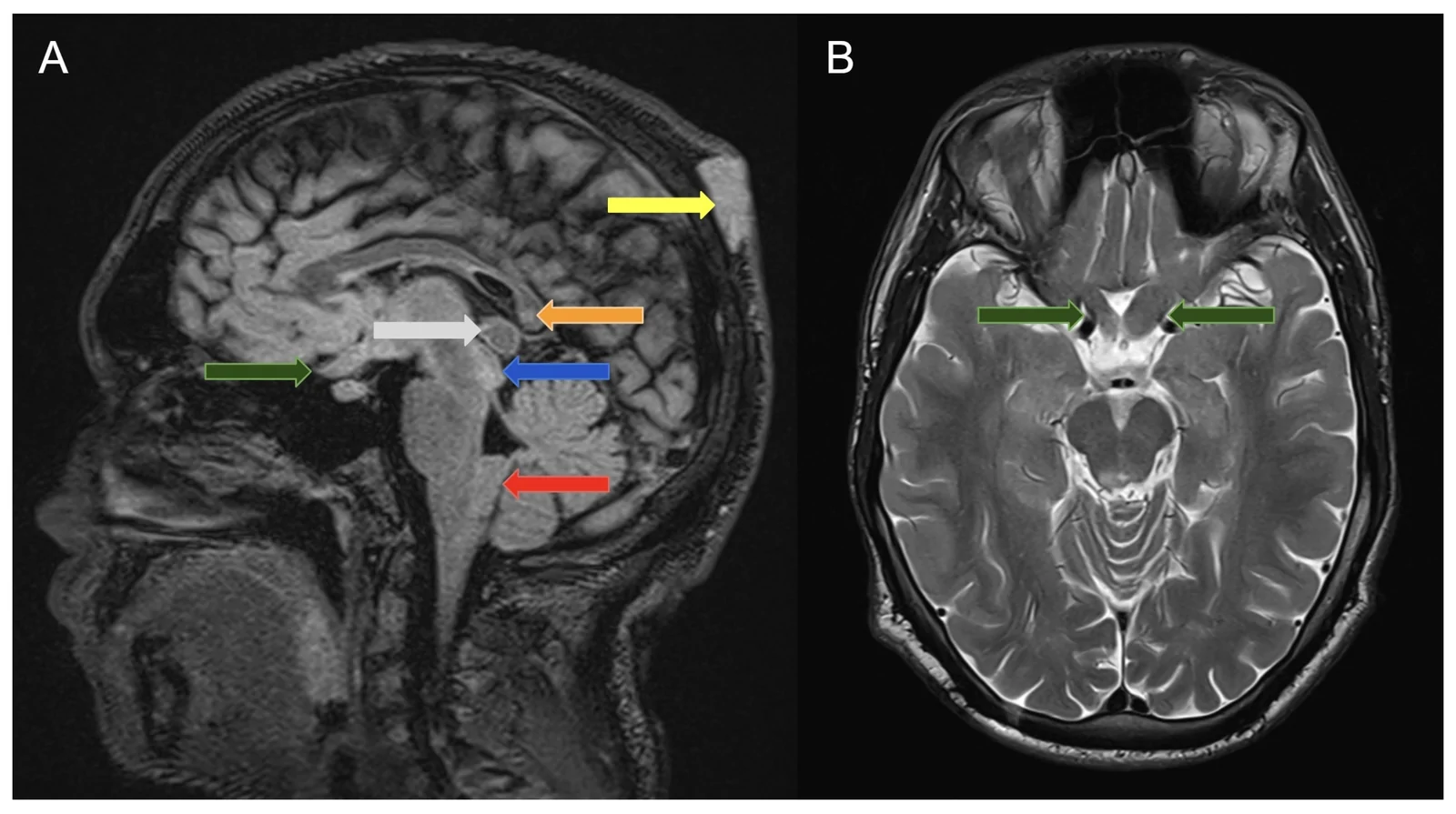
(**A**) Sagittal FLAIR MRI demonstrating multiple hyperintense intracranial lesions, including cavernoma in the medulla oblongata (red arrow), focal abnormal signal intensity (FASI) within the right splenium of the corpus callosum (orange arrow), gliomatous lesion involving the tectal plate/inferior collicular region (blue arrow) with occlusion of the cerebral aqueduct, optic pathway glioma (green arrow) and neurofibroma (yellow arrow) on the surface of the skin and subcutaneously in the scalp area. In the image there is also a pineal cyst (white arrow). (**B**) Axial T2-weighted image demonstrating bilateral optic nerve thickening consistent with prechiasmatic optic nerve gliomas (green arrows), left-sided predominance evident, approximately 6 mm on the right side and 9 mm on the left side.

**Figure 3:**
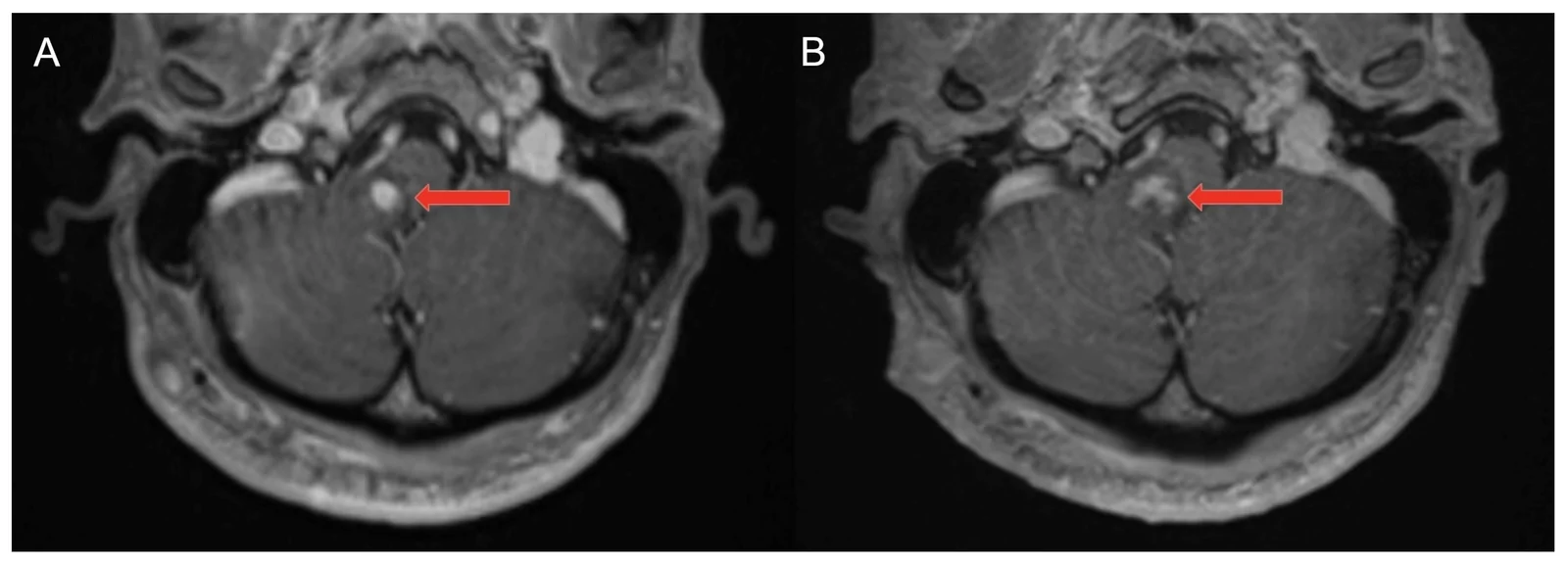
Axial post-contrast T1-weighted MRI images obtained in 2023 (**A**) and 2025 (**B**) showing enlargement of the dorsal right brainstem cavernoma (red arrows) from approximately 13 mm to 17 mm, with heterogeneous contrast enhancement, initially raising suspicion for brainstem glioma.

**Figure 4:**
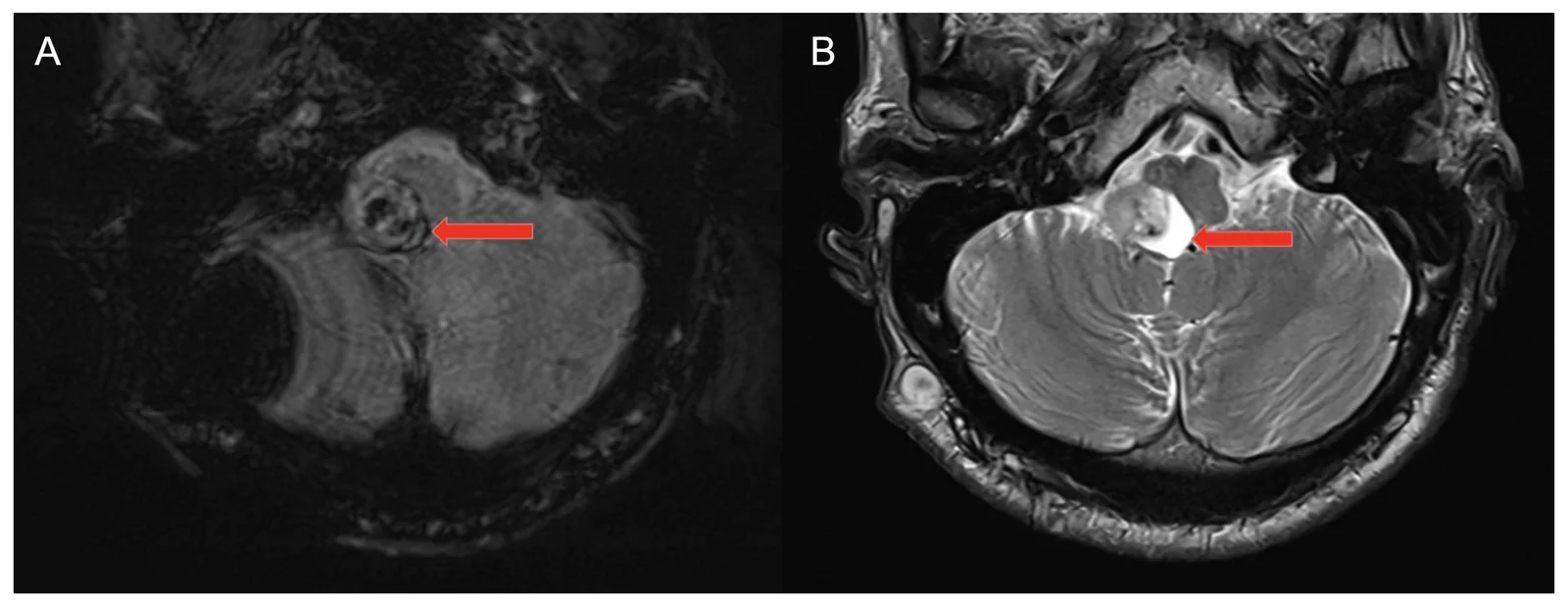
(**A**) Axial SWI MRI revealing marked susceptibility blooming within the brainstem lesion corresponding to hemosiderin deposition (red arrow). Imaging artifact associated with prior trepanation of the right occipital bone regarding the implantation of ventriculoperitoneal shunt. (**B**) Axial T2-weighted MRI demonstrating a cavernoma in medulla oblongata with an associated cyst, causing compression of the fourth ventricle (red arrow).

**Figure 5:**
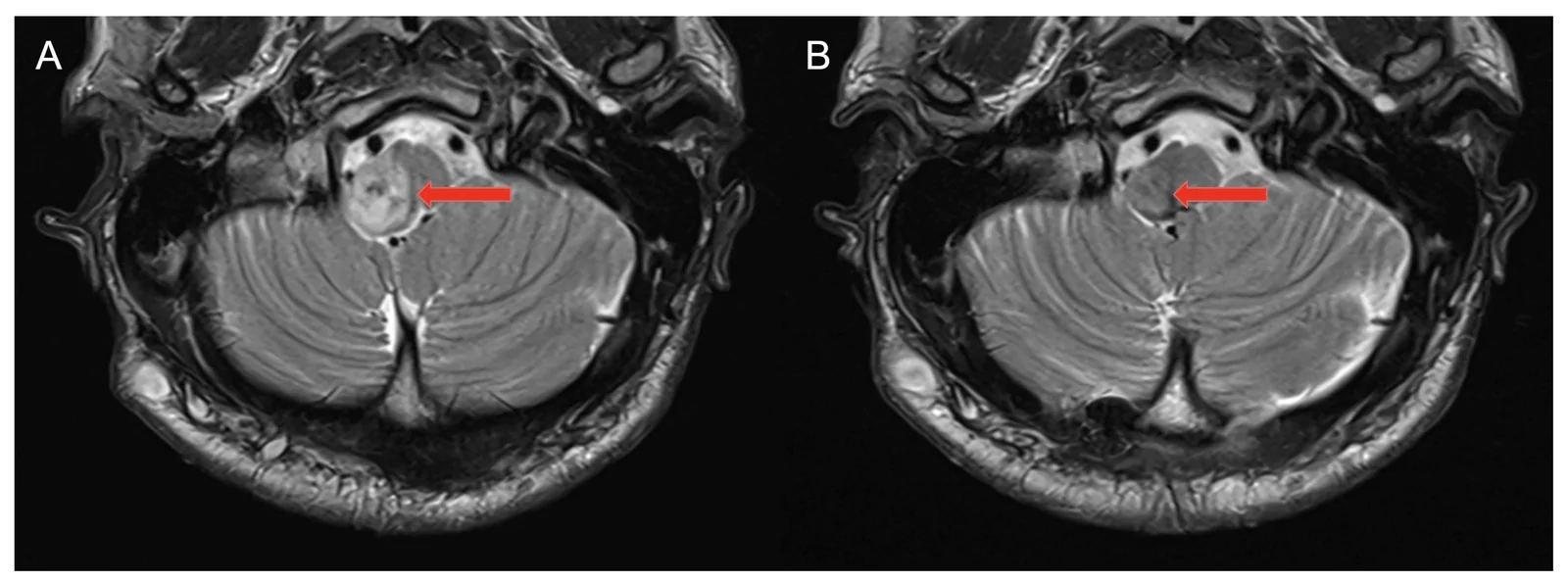
(**A**) Axial T2-weighted MRI obtained preoperatively in 2025 showing a lesion of the medulla oblongata (red arrow) that initially raised suspicion for glioma but postoperatively was confirmed as cavernoma. (**B**) Axial T2-weighted MRI obtained postoperatively in 2026 following surgical resection of the cavernoma and associated cyst; residual cavernomatous tissue measuring approximately 1 cm still persisted (red arrow).

**Figure 6:**
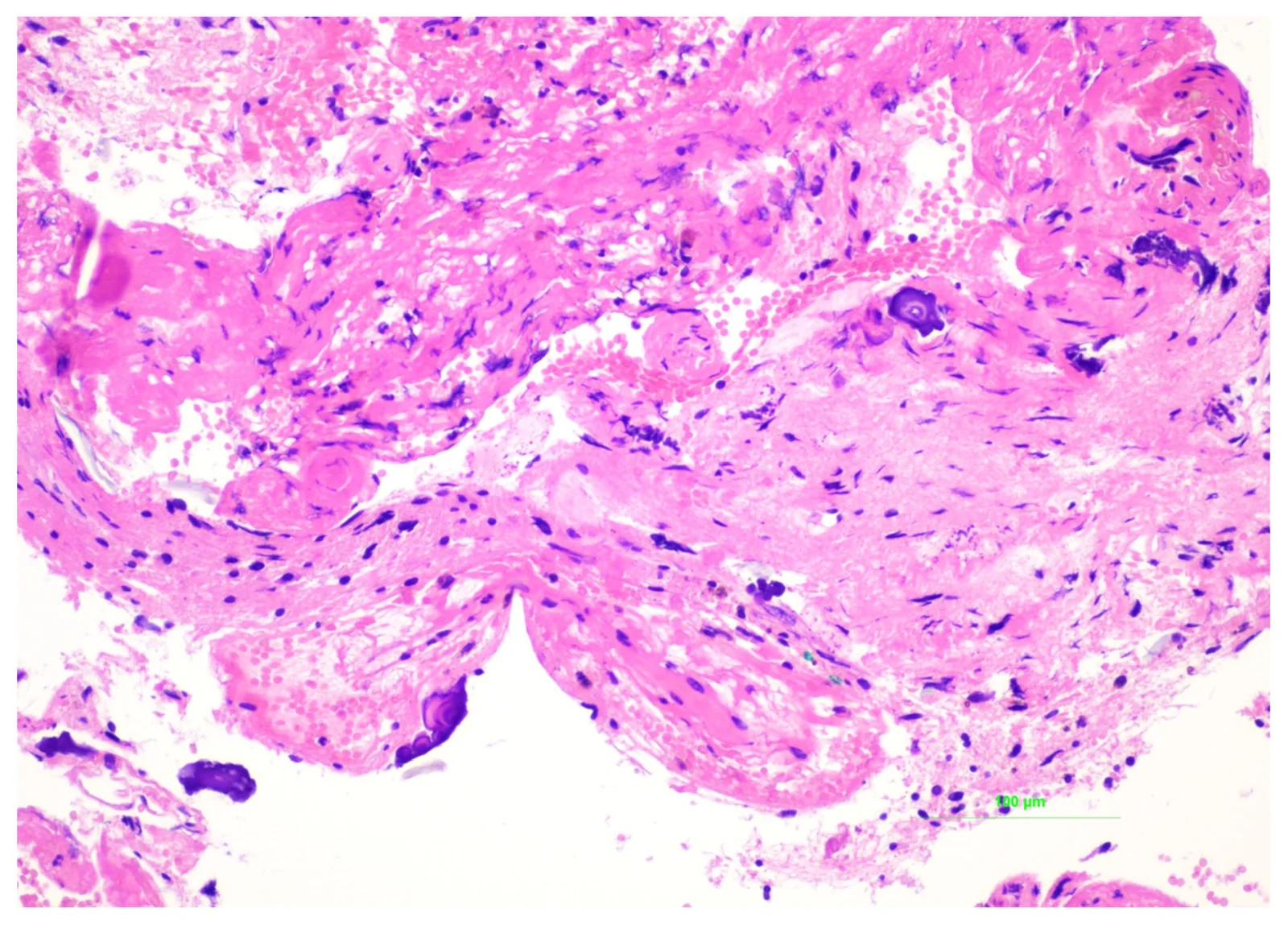
The general structure of the surgically removed pathological tissues, showing a cluster of collapsed blood vessels lined by plain endothelium. The vascular walls feature microcalcifications and focal hyalinization but lack a properly organized smooth muscle layer. Visualization by hematoxylin–eosin stain, original magnification 200×.

**Figure 7:**
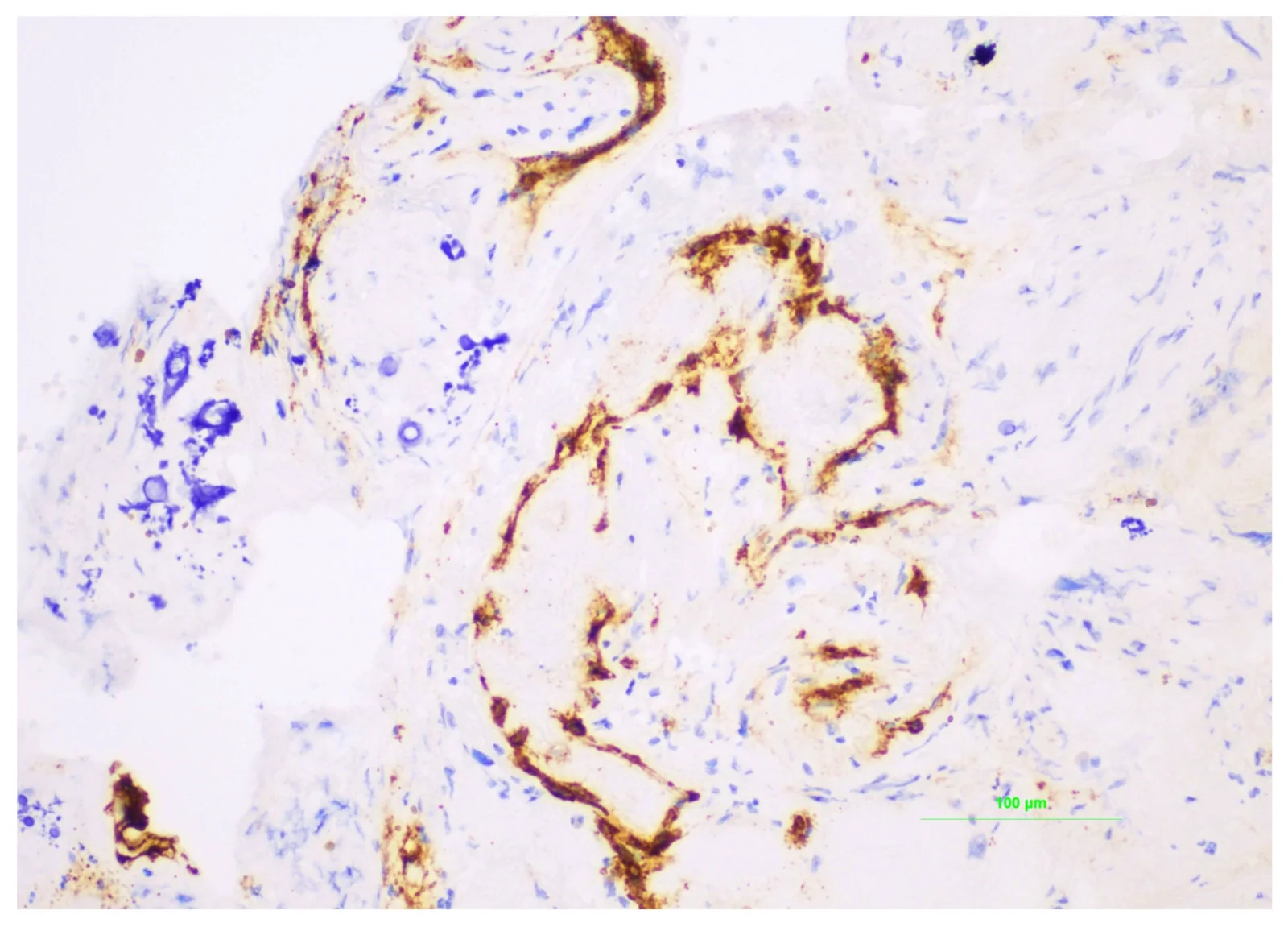
Immunohistochemical staining for CD34 highlights the endothelial lining of the dilated vascular channels and supports their vascular nature. Immunoperoxidase, anti-CD34, original magnification 200×.

**Figure 8:**
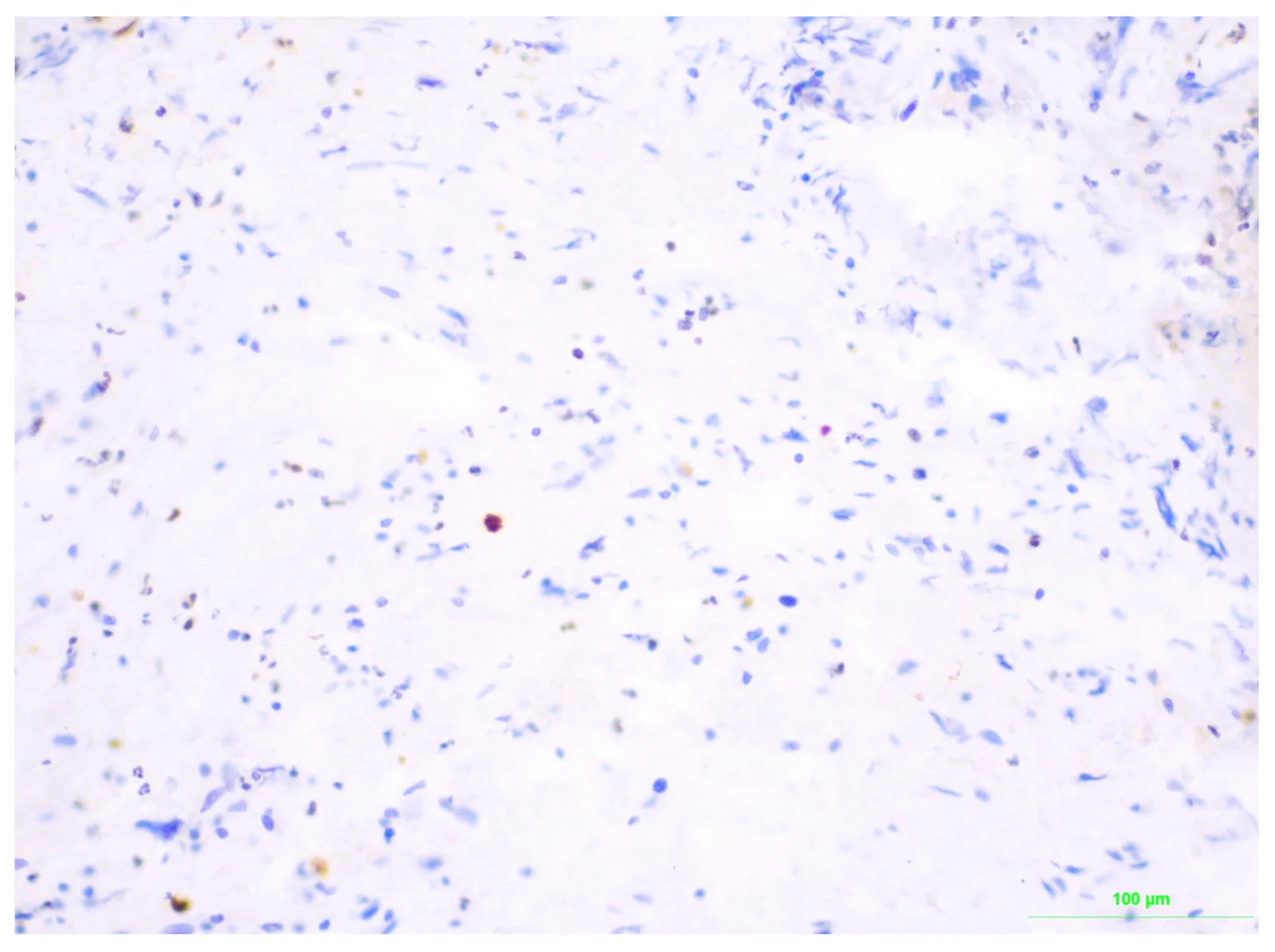
Low proliferation activity: Ki-67 proliferation index is less than 1% indicating low proliferative activity in the examined tissue. Immunoperoxidase, anti-Ki-67, original magnification 200×.

**Figure 9:**
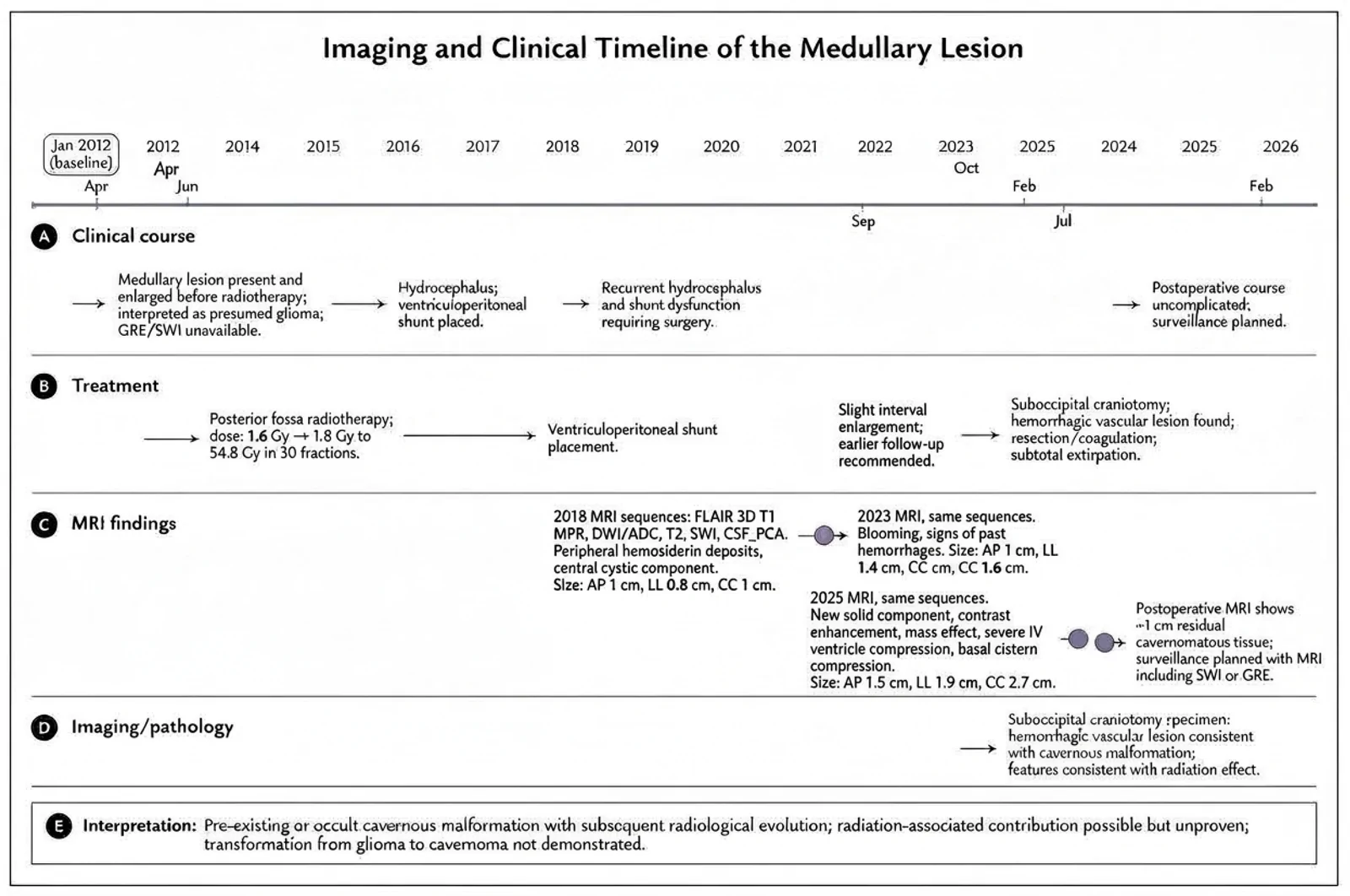
Chronological summary of the clinical course, treatment, MRI findings, and diagnostic interpretation of the right medullary lesion.

## Data Availability

The original contributions presented in this study are included in the article. Further inquiries can be directed to the corresponding author. Dataset available on request from the authors.
